# Neural Oscillations Track Subjective and Pupillary Arousal During Naturalistic Movie Viewing

**DOI:** 10.1111/ejn.70543

**Published:** 2026-05-12

**Authors:** Magdalena Camenzind, Melanni Nanni‐Zepeda, Anna P. Giron, Konrad Dapper, Michael Esterman, Flavio Frohlich, Agnieszka Zuberer

**Affiliations:** ^1^ Department of Psychiatry University of North Carolina at Chapel Hill Chapel Hill North Carolina USA; ^2^ Carolina Center for Neurostimulation University of North Carolina at Chapel Hill Chapel Hill North Carolina USA; ^3^ Department of Psychiatry and Psychotherapy, Tübingen Center for Mental Health (TüCMH), University of Tübingen Tübingen Germany; ^4^ German Center for Mental Health (DZPG), partner site Tübingen Tübingen Germany; ^5^ Department of Biology Technical University Darmstadt Darmstadt Germany; ^6^ MEG‐Center University of Tübingen Tübingen Germany; ^7^ Department of Psychology University of Western Ontario London Ontario Canada; ^8^ National Center for PTSD VA Boston Healthcare System Boston Massachusetts USA; ^9^ Department of Psychiatry Boston University Chobanian and Avedisian School of Medicine Boston Massachusetts USA

**Keywords:** arousal, electroencephalography (EEG), emotion, naturalistic stimuli, neural oscillations, Pupillometry

## Abstract

Movies evoke dynamic emotional experiences that fluctuate moment‐to‐moment. While fMRI has mapped these fluctuations, the real‐time continuous oscillatory dynamics underlying naturalistic viewing remain less understood. In this study, 25 adults watched an emotionally rich short film while EEG, continuous subjective arousal annotations and pupil diameter were recorded. Inter‐subject correlation (ISC) analyses revealed robust synchronization in behavioural and pupillary arousal across the cohort. Leveraging these shared, group‐level arousal trajectories to probe individual‐level cortical processing, we mapped the neural networks correlating with these two arousal signals. Both pupillary and subjective arousal negatively correlated with low‐frequency power in occipitoparietal regions, reflecting bottom‐up sensory gain control and attentional gating. Furthermore, high‐arousal epochs were marked by low‐frequency desynchronization in the precuneus; this cortical activation likely indexes the rapid retrieval of episodic memories required to update the viewer's situational model during plot shifts. Finally, while both measures tracked low‐frequency acoustic features in the auditory cortex, subjective arousal was more prominently associated with extended top‐down semantic networks and central theta activity. These findings highlight that while pupillary arousal heavily reflects bottom‐up sensory intensity, subjective reporting captures active cognitive integration. Together, this demonstrates how emotional arousal acts as a dynamic control signal, orchestrating a complex interplay of sensory gating, memory updating and top‐down evaluation to make sense of the unfolding narrative.

## Graphical abstract

In this study, participants watched an emotionally evocative movie while EEG, pupil size and continuous subjective arousal annotations were recorded. By extracting the mean group arousal signatures, we revealed a cross‐modal convergence (*r* = 0.40) between the pupillary and subjective measures. Each of these continuous arousal timeseries was then independently correlated over time with low‐frequency EEG power. Crucially, source‐level analyses revealed characteristic network profiles: while pupillary and subjective arousal both reflect bottom‐up sensory gating within attentional networks, subjective arousal prominently engages higher‐order systems, including the Default Mode Network and fronto‐central regions, to support the active cognitive integration of the unfolding narrative.

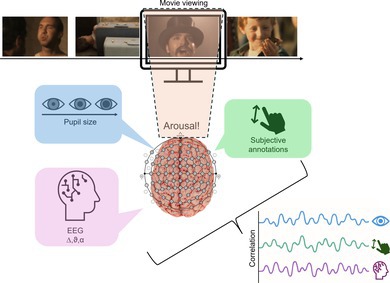

**Abbreviations**
AI: artificial intelligence
ASR: artefact subspace reconstruction
BEM: boundary element method
DAN: dorsal attention network
DMN: default mode network
EEG: electroencephalogram
ERP: event‐related potential
FDR: false discovery rate
FOOOF: fitting oscillations & one over f
IAF: individual alpha frequency
ISC: inter‐subject correlation
MNE: minimum‐norm estimation
MN imaging: minimum‐norm imaging
MRI: magnetic resonance imaging
MTG: middle temporal gyrus
PC: personal computer
PSD: power spectral density
RGB: red green blue
SD: standard deviation
SMG: supramarginal gyrus
SNR: signal‐to‐noise ratio
VAN: ventral attention network

## 1 Introduction

Emotions are linked to a multifaceted response system, involving subjective experience, peripheral and central physiology and behaviour. Arousal can take various forms (e.g., emotional, autonomic, sexual; Russell 1980) that share some psychological (e.g., increased emotional reactivity; Pfaff et al. 2012) and physiological (e.g., sympathetic activation) features. Emotional arousal refers to the degree of intensity or activation linked with an emotional state. It plays a central role in shaping emotional experiences by influencing which competing mental representations gain strength: specifically, arousal biases cognition by amplifying attention toward highly salient or goal‐relevant stimuli while suppressing less relevant background details (Mather and Sutherland 2011). Studies suggest that there is little evidence for neural circuitries supporting specific emotional categories but instead suggest that emotions exist along a continuous spectrum of affective experiences, characterized by variations in arousal and valence (Wilson‐Mendenhall et al. 2013), fluctuating dynamically in response to external stimuli over minutes (Kuppens et al. 2010) or even seconds (Mikutta et al. 2012). Naturalistic stimuli such as movies provide an ecologically valid means to study emotional fluctuations in a way that mimics real‐world experiences (Koenig et al. 2008; Saarimäki 2021). However, explicitly probing these emotional experiences in real‐time can disrupt or alter their natural expression. Further, analysing continuous data is inherently challenging due to the absence of clear event boundaries and the complex nature of emotional changes in naturalistic scenarios (Sonkusare et al. 2019). Consequently, it remains poorly understood whether subjective emotional experiences and autonomic physiological responses share common underlying neural substrates during these dynamic events, a gap this study aims to bridge.

Previous research has demonstrated that naturalistic stimuli such as music, virtual reality and movies can dynamically modulate subjective emotional arousal and related brain oscillations. For example, subjective arousal during music listening was associated with decreased alpha power over right‐frontal regions (Mikutta et al. 2012), while subjective arousal during a virtual roller coaster was linked to reduced alpha power over parieto‐occipital regions (Hofmann et al. 2021). Together, this indicates that alpha reductions may reflect local modulations of cortical engagement during emotionally intense moments in time. Studies combining functional magnetic resonance imaging and subjective annotations of arousal during movie viewing further found that emotionally charged movie scenes led to increased inter‐subject synchronization of brain activity, particularly in emotion‐related and attention networks (Nanni‐Zepeda et al. 2024), as well as in somatosensory and visual cortices (Nummenmaa et al. 2012). These findings suggest that emotional arousal shapes large‐scale brain dynamics, supporting shared processing of emotionally salient events.

Continuous subjective annotations during movie viewing are a powerful approach in tracking subjective arousal changes from moment to moment. Often, they are collected during a second viewing of the stimulus (retroactive) (Dmochowski et al. 2012; Hofmann et al. 2021; Hutcherson et al. 2005; Nummenmaa et al. 2012), since annotating in real time can interfere with natural arousal responses (Lieberman et al. 2007). These retroactive subjective annotations, however, carry the risk that individuals may systematically misremember their original experience. In contrast, autonomic markers such as pupil diameter (Bradley et al. 2008; Wang et al. 2018) and skin conductance (Bach et al. 2010; Eisenbarth et al. 2024) can be taken unobtrusively. Indeed, pupil diameter may provide a seamless real‐time marker of arousal, reflecting the balance between sympathetic activation (via norepinephrine‐driven dilation) and parasympathetic control (via acetylcholine‐mediated constriction; Hasselmo and Sarter 2011; Jones 2003, 2004; Meissner et al. 2024). Pupil diameter recordings during the actual movie experience may provide direct read‐outs of arousal circumventing the retroactive difficulty. However, little is understood about how body signals (e.g., heart rate, skin conductance, somatic activation) align with dynamic subjective experiences during simulated naturalistic scenarios such as movie viewing.

According to a componential model of emotion, emotional responses span experiential, physiological and behavioural domains, each contributing uniquely to an emotional episode. Indeed, Mauss et al. (2005) found that while subjective experience and behaviour were strongly linked, physiological responses showed only modest associations with these other domains. Notably, greater coherence across all three domains emerged during intense amusement but not during sadness. Within this componential framework, pupil diameter provides a window onto the physiological component of emotion. Converging evidence shows that emotional arousal reliably increases pupil size, across visual and auditory modalities and independent of valence and that these pupillary changes covary with sympathetic activation and with subjective ratings of emotional intensity (Bradley et al. 2008; Kinner et al. 2017; Nakakoga et al. 2020). Building on this work, Kang and Wheatley (2017) demonstrated that moment‐to‐moment emotional salience in narratives is mirrored in collective pupillary synchrony across individuals, suggesting that shared pupillary dynamics can index shared emotional engagement over time.

Recent work suggests that engagement and arousal modulate not just brain oscillations but also the aperiodic background (1/f slope) of the power spectrum, reflecting shifts in the cortical excitation/inhibition balance (Gao et al. 2017; Kałamała et al. 2024). While acute arousal typically flattens the slope (Lendner et al. 2020), complex naturalistic states like immersion may involve distinct spectral shifts related to the suppression of internal noise or focused attention (Gyurkovics et al. 2022; Kałamała et al. 2024). Furthermore, slow brain oscillations are especially well suited to interact with bodily rhythms because both operate on similar temporal scales. Bodily signals such as heart rate, respiration and gastric rhythms typically unfold in the low‐frequency range (e.g., < 1 to ~10 Hz), overlapping with delta, theta and alpha band activity in the brain (Xiong and Garfinkel 2023). This frequency compatibility facilitates phase alignment and entrainment, enabling effective brain–body coupling. Within the brain, oscillatory communication is organized hierarchically, with slower rhythms modulating and coordinating faster ones across spatial and temporal scales (Hamill 2023; Klimesch 2013, 2018). Efficient coupling within this hierarchy, such as phase–amplitude (Canolty and Knight 2010) and phase–phase interactions (Palva et al. 2005, 2010; Tass et al. 1998), is often strongest when the interacting frequencies maintain low‐integer (harmonic) ratios. These harmonic relationships enable slower oscillations to act as a scaffolding for faster dynamics, positioning them as a bridge between the slow timescale of bodily physiology and the faster computations of the brain. Overall, this suggests that slow neural rhythms provide an interface for brain–body integration and may serve as a direct neural correlate of physiological arousal, also measurable via indices such as pupil diameter.

Delta oscillations (1–4 Hz) are fundamentally linked to biologically primitive mechanisms of salience detection and motivation. Evolutionarily associated with survival, delta activity prioritizes the processing of motivationally significant stimuli, such as threats, rewards or pain, facilitating their detection even at subconscious levels (G. Knyazev et al. 2009; G. G. Knyazev 2007; Parnefjord and Başar 1999; Polich and Kok 1995). Theta oscillations (4–7 Hz) typically reflect the operation of the Behavioural Inhibition System (BIS) and the regulation of emotional states (G. G. Knyazev 2007). Functionally, theta activity mediates communication between subcortical limbic regions and executive cortical networks to support conflict monitoring (Cavanagh and Frank 2014). Furthermore, recent evidence links theta activity directly to physiological arousal, with fluctuations in pupil diameter, a proxy for noradrenergic activity, predicting increased parietal theta power during states of heightened arousal (Stitt et al. 2018). Finally, alpha oscillations (8–12 Hz) act as a sensory gating mechanism, where high power reflects the functional inhibition of task‐irrelevant regions to protect internal processing (Klimesch 2012). While alpha suppression typically signals active cortical engagement and is often observed during states of high emotional arousal (De Cesarei and Codispoti 2011), this modulation is context‐dependent. Alpha activity is flexibly adjusted to support goal‐directed processing: power decreases in relevant sensory areas to facilitate intake, while increasing in irrelevant regions to filter distraction or regulate affective interference (Jensen and Mazaheri 2010; Uusberg et al. 2013).

Taken together, there are indications that slow oscillations in the delta to lower alpha range may link to moment‐to‐moment arousal fluctuations. Despite extensive research on arousal‐related neural oscillations, it remains poorly understood whether subjective and physiological arousal markers share common neural substrates during naturalistic emotional experiences. A key challenge is that ecological scenarios are less controlled, and individual responses may capture considerable idiosyncrasies, obscuring common patterns. Here, we calculate the average of arousal fluctuations across participants to identify dominant patterns and assess how these shared components relate to oscillatory changes during movie viewing.

### 1.1 Aims

Our primary aim was to identify the oscillatory correlates of continuous, moment‐to‐moment changes in physiological (pupil diameter) and subjective (arousal annotations) emotional arousal during the viewing of a highly engaging short film. We focused on continuous spectral power because, unlike static or event‐related measures, it provides a time‐resolved window into dynamic neural fluctuations as they unfold over the course of a narrative. To characterize the global neurophysiological state induced by the stimulus, we first compared the aperiodic (1/f) slope and periodic activity between resting‐state and movie‐watching. Next, to address the high inter‐subject variability inherent to EEG topography, we adopted a mixed‐level, stimulus‐driven analytical approach. Because compelling narratives elicit synchronized experiences across an audience, we expected high inter‐subject correlation (ISC) in both the subjective annotations and pupil diameter. By extracting the group‐level mean of these measures, we could filter out idiosyncratic individual noise and isolate a robust, shared arousal trajectory driven purely by the movie. We then probed moment‐to‐moment modulations in individual‐level brain oscillations by correlating them directly with this shared group‐level signal.

Based on prior research linking low‐frequency oscillations to affective processes, we formulated specific regional hypotheses. Drawing on evidence that frontal alpha activity (Allen et al. 2004; Heller et al. 1997; Mikutta et al. 2012) and parieto‐frontal theta activity (Aftanas and Golocheikine 2001; Stitt et al. 2018; Suetsugi et al. 2000) track emotional intensity, we hypothesized that right frontal alpha power and parietal theta power would significantly correlate with the shared arousal trajectories induced by the narrative.

## 2 Methods

### 2.1 Participants

We collected data from 47 participants at the Attention and Affect Laboratory within the Department of Psychiatry and Psychotherapy at the University of Tübingen, Germany. Eight participants did not give consent for international data processing, resulting in a sample of 39 participants (27 females, mean age = 35 ± 15.8). All participants voluntarily took part in the study, reported no history of neurological or psychiatric disorders, provided written informed consent and received monetary compensation. This study was conducted in accordance with the Declaration of Helsinki and approved by the ethics committee of the Medical Faculty of the University of Tübingen. All participants provided written informed consent after receiving detailed study information.

Within these 39 participants, 35 participants had continuous subjective annotations (25 females, mean age = 34.4, SD = 15.7) and 30 participants (22 females, mean age = 32.6, SD = 15.1) had valid pupil data. Valid pupil data are defined as having continuous recording points throughout the entire session, whereas data with missing time segments (minutes) were classified as invalid. Data from these participants were used to compute the group‐level arousal and pupillary trajectories, which were then correlated with individual‐level EEG activity.

EEG recordings were obtained from 32 participants, with 7 datasets excluded during pre‐processing due to poor signal quality, defined as excessive noise, persistent artefacts or an overall low signal‐to‐noise ratio that precluded reliable analysis (see Section 2.5: EEG recording and pre‐processing). Consequently, a total of 25 EEG datasets (21 females, mean age = 33.4, SD = 15.6) were included in the final brain‐behaviour analysis.

### 2.2 Experimental Paradigm

The data presented here were collected as part of a larger experiment involving EEG recordings during the performance of an attention task, resting state recordings and movie viewing (in that order). Here, we focus on analyses of the movie data set. During this part, participants underwent three sequential conditions. First, participants were instructed to maintain a passive resting state for 2 min. Following this, they watched a 20‐min emotionally evocative film, The Butterfly Circus (Joshua Weigel 2009, Peacetree Productions, United States) with original audio, while positioned on a chin rest to minimize head movement. Pupillometry data were simultaneously recorded throughout the session, whereas subjective emotional arousal annotations were collected while re‐watching the movie clip 5–10 min after the EEG session. The Butterfly Circus depicts the journey of a limbless individual who, through perseverance and self‐discovery, finds meaning and purpose within a travelling circus. The film is known to elicit a wide range of emotions with varying intensities and has been utilized in prior research to investigate brain dynamics associated with perceptual and emotional processing (van der Meer et al. 2020; Nguyen et al. 2017).

### 2.3 Arousal Annotations

Approximately 5–10 min after the EEG session, participants rewatched the movie clip while providing continuous emotional arousal annotations reflecting their experiences during the initial viewing. Continuous emotional arousal annotations were collected using the CARMA software (Girard 2014). Specifically, participants used a continuous rating scale ranging from 0 (‘not at all’) to 250 (‘very much’) to indicate the perceived emotional arousal during movie watching, recalling their initial viewing experience. The scale was displayed adjacent to the video playback, and arrows on the keyboard were used to adjust the bar in the visual analog scale. The instruction given to participants was: ‘Following is a short rating of the movie clip that you previously watched. You will see the movie clip again on the PC screen and are asked to rate how emotional you feel while watching’. Annotations were sampled at 1 Hz.

### 2.4 Pupil Data Acquisition and Pre‐Processing

Pupillometry data, specifically pupil size, were recorded during the first movie‐viewing session using a Tobii Pro Nano (60 Hz) eye‐tracking system, with a sampling rate of 60 Hz from the left eye. For the duration of the movie, the participants were seated approximately 60 cm from the monitor and rested their heads on a chinrest to reduce movement artefacts in the pupil data as well as in the EEG data.

Pupil size data underwent a custom multi‐step preprocessing pipeline. The first 300 time points were excluded to minimize luminance adaptation effects (Belliveau et al. 2023). Signal loss and device malfunctions were addressed by removing large spikes, and missing data were interpolated using a 100‐point moving median. A de‐blinking procedure removed data points with first derivatives exceeding 3 standard deviations. We applied a 100‐point one dimensional median filter to the signal to reduce noise while preserving larger‐scale trends. Subjects with > 40% missing data at any stage were excluded from analysis.

To standardize the length of all time series and align them with the subjective annotations, we resampled each participant's data to a 1 Hz resolution (one sample per second). Specifically, we generated a uniform time grid matching the movie's duration in seconds and used MATLAB's interp1 function to linearly interpolate the preprocessed signals onto this new grid. To minimize the influence of luminance on pupil size fluctuations, we first derived luminance values from the movie video by converting RGB frames to grayscale images and computing the mean intensity in grey levels for each frame. This luminance time series was then downsampled to match the one‐second resolution of the pupil data. We then used a linear regression to regress out luminance from the individual preprocessed pupil time courses. After luminance removal, a Gaussian filter with a moving window of 40 time points was applied to smooth the pupil data for each participant.

### 2.5 EEG Recording and Pre‐Processing

The data presented here were collected within a larger study involving attention task assessments and a movie viewing paradigm. Here, we report only analyses on the movie EEG data and eyes‐open resting state EEG data (2 min) recorded before movie onset. EEG data were recorded during the movie viewing (20 min), using a 64‐channel EEG cap from g.tec, medical engineering GmbH (Scheidlberg, Austria) employing the international extended 10–20 system for electrode placement. This configuration provides standardized spatial coverage of cortical activity, ensuring consistent and reproducible electrode positioning across participants. The ground electrode was placed at AFz, while the physical reference electrode was attached to either the left or right earlobe depending on participant comfort. While this initial physical setup is lateralized, offline global average re‐referencing was subsequently applied across all 64 channels. This step successfully removed common‐mode artefacts, neutralized the initial single‐sided reference bias and created a reference‐free montage for all subsequent signal analyses.

EEG data were preprocessed semi‐automatically, using custom scripts written with MATLAB (R2023a, MathWorks, Natick, MA) and EEGLAB (v2023; Delorme and Makeig 2004). A zero‐phase 4th‐order Butterworth high‐pass filter with a cutoff at 1 Hz was applied, followed by artefact subspace reconstruction (ASR) to eliminate high‐variance noise and reconstruct missing segments (Mullen et al. 2013). This method also identified noisy channels, which were after visual inspection replaced using spherical spline interpolation based on neighbouring electrodes. On average, 1.16 ± 1.34 noisy channels were interpolated per participant (range: 0–4 channels). Furthermore, power line artefacts at 50 Hz were automatically removed using the ZapLine+ algorithm (de Cheveigné 2020; Klug and Kloosterman 2022) and data was down sampled to 200 Hz to reduce computational load while maintaining sufficient temporal resolution. A global average re‐reference was applied to mitigate residual noise and enhance signal consistency across channels, leveraging the 64 electrodes to approximate a spherical head model. A semi‐automated approach of Independent Component Analysis (ICA) was conducted using EEGlab's ICAlabel algorithm to further isolate and remove artefacts. Specifically, independent components labelled muscle, eye, heart, channel noise and line noise were rejected if they surpassed a probability threshold of 0.7 (indicating 70% confidence by the algorithm). These automatic classifications were subsequently verified by visual inspection. Importantly, all components categorized as ‘brain’ or ‘other’ were retained and not automatically removed (Jung et al. 2000). For the final sample of 25 participants, an average of 9.31 ± 3.09 artifactual components were rejected (range: 3–14). Following ICA, the overall quality of each dataset was objectively assessed by evaluating the remaining spatial rank of the data. This final rank reflects the total spatial degrees of freedom remaining after accounting for both channel interpolation and component rejection. Because subsequent source localization requires sufficient spatial degrees of freedom to generate stable inverse solutions, an a priori threshold was set to retain a minimum data rank of 48 (out of 64 channels, preserving ≥ 75% of the spatial variance). Datasets falling below this rank were considered to have an unacceptably low signal‐to‐noise ratio and were excluded from further analysis. Based on this threshold, 7 datasets were excluded, resulting in a final sample of 25 participants (see Figure S1 for a visualization of retained components per participant).

### 2.6 ISC

Before assessing how individual differences in arousal modulate brain activity, we first sought to confirm that the movie stimulus successfully elicited reliable, time‐locked neural responses across the cohort. ISC is ideal for this validation, as it isolates stimulus‐driven neural responses shared across participants without requiring explicit event markers (Finn et al. 2020; Hasson et al. 2004; Nastase et al. 2019; Simony et al. 2016). Compared to approaches based on General Linear Models, ISC is data‐driven and can detect meaningful shared variance even when the most relevant stimulus features are unknown or difficult to model (Hejnar et al. 2007; Pajula et al. 2012). ISC was computed to quantify the synchronization of both behavioural and neural time series across the cohort. For the subjective arousal (*N* = 35) and pupil diameter (*N* = 30) data, Pearson correlation coefficients were calculated between all unique participant pairs. To ensure mathematical validity, all pairwise *r*‐values were Fisher z‐transformed prior to averaging and group‐level evaluation. To determine the statistical significance of this group‐level ISC, a nonparametric circular time‐shifting permutation test was employed. Each participant's time series was shifted by a random temporal lag to disrupt synchronization to the cinematic stimulus while preserving the inherent autocorrelation of the signal. This process was repeated 1000 times to generate a null distribution of surrogate ISC values, from which the empirical *p*‐value was derived (analysis code available on OSF: https://osf.io/75mzh/overview). For the EEG data (*N* = 25), to sidestep phase‐mismatch artefacts, the ISC was computed on the slow amplitude fluctuations (power envelopes) rather than raw oscillatory phases. Specifically, Pearson correlation coefficients were calculated between all unique participant pairs using the continuous time courses of mean spectral power (the extraction of which is detailed in Section 2.9) separately for the delta [2–4 Hz], theta [4–7 Hz] and low IAF bands at each channel. Finally, these averaged ISC values were visualized using topographical scalp plots to examine the spatial distribution and magnitude of neural synchronization.

### 2.7 Spectral Decomposition and Parametrization

Neural power spectra contain both oscillatory peaks (periodic rhythms) and aperiodic (1/f) background activity. Since states of high arousal can flatten the aperiodic slope and potentially confound estimates of narrowband oscillatory power, we employed spectral parametrization to mathematically separate these components.

#### 2.7.1 Data Selection and Preprocessing

To ensure direct comparability between conditions and control for signal‐to‐noise ratio, spectral analyses were restricted to 2 min of data for each condition. Specifically, the full 2‐min eyes‐open resting‐state recording was compared against the first 2 min of the movie‐watching period.

#### 2.7.2 Spectral Decomposition

We utilized the SpecParam algorithm (v1.1.0; formerly FOOOF) (Donoghue et al. 2020) to isolate oscillatory peaks from the broadband 1/f background. This algorithm models the power spectrum (S) as a linear combination of an aperiodic component (L) and N Gaussian peaks (G_n_):

$$S\left(f\right)=L\left(f\right)+\sum_{n=1}^{N}G_{n}\left(f\right)$$

For general spectral characterization (aperiodic exponent and flattened spectra), Power Spectral Densities (PSDs) were calculated using Welch's method (200 Hz sampling rate, 1.28 s Hanning window, 50% overlap).

#### 2.7.3 Regional Specificity

To generate representative spectra for plotting and validation, PSDs were calculated for individual electrodes and then averaged across channels to create a single pooled spectrum for the Occipital (PO3, PO4, PO7, PO8, POz, O1, O2, Oz) and Fronto‐Central (FPz, FP1, FP2, AFz, AF3, AF4, AF7, AF8, Fz, F1–8, FCz, FC1‐FC8, Cz, C1–8) regions.

#### 2.7.4 Global Field Power

For aperiodic exponent analysis, a global average PSD was computed across all electrodes.

The SpecParam model was fit to the power spectrum over the 2–40 Hz range in ‘fixed’ aperiodic mode. Settings included a peak width limit of 1–20 Hz, a maximum of 6 peaks and a minimum peak height of 0.15 log10 units.

#### 2.7.5 Derived Spectral Metrics

Based on the decomposition above, we extracted three key metrics:

Aperiodic Exponent (χ):

To characterize global shifts in the neural baseline, we extracted the aperiodic exponent (representing the 1/f slope) from the global field power spectrum. The exponents were compared between the Resting State (2 min) and Movie Watching (first 2 min) conditions using a paired‐samples *t*‐test (alpha = 0.05).

Flattened Spectra:

To visualize and statistically compare oscillatory activity independent of the 1/f background, we subtracted the fitted aperiodic component from the original power spectrum (S(f)–L(f)). We generated double plots for the averaged Occipital and Fronto‐Central spectra to inspect periodic signals relative to the baseline.

Individual Alpha Frequency (IAF):

The alpha band was specifically restricted to the lower Individual Alpha Frequency (IAF‐1 to IAF). The rationale for targeting low IAF rather than the broad alpha band is twofold: functionally, lower alpha rhythms are strongly associated with broad changes in general arousal and sustained attention, whereas upper alpha relates more to task‐specific cognitive processing (Klimesch et al. 1999). Furthermore, the slower temporal scale of the low IAF band (< 10 Hz) aligns more closely with the slow, continuous dynamics of the physiological bodily signals (e.g., pupillary arousal) investigated in this study (Xiong and Garfinkel 2023). IAF was determined using the 2‐min resting‐state data. To achieve finer frequency resolution for peak detection, the PSD calculation was modified for this specific metric: we used a 2‐s Hann window zero‐padded to 10 s (resulting in 0.1 Hz resolution; similar in Zhang et al. 2022). The SpecParam algorithm was applied to the global average spectrum (1–40 Hz range). The IAF was defined as the peak frequency with the highest power within the 8–13 Hz range of the flattened spectrum. Low‐IAF was defined as the range IAF‐1 to IAF. This method was chosen to minimize potential artefacts that could arise from edge effects or residual aperiodic components, ensuring a more accurate identification of the true alpha peak.

To identify frequency‐specific differences in oscillatory power between the Resting State and Movie Watching conditions, we performed nonparametric cluster‐based permutation *t*‐tests (Maris and Oostenveld 2007) on the flattened spectra. This analysis was conducted separately for the averaged Occipital and Fronto‐Central regions. We compared the spectral power at each frequency bin (2–18 Hz). Clusters were formed by grouping adjacent frequency bins where the observed t‐statistic exceeded the critical threshold corresponding to alpha = 0.05 (uncorrected). The cluster‐level statistic was defined as the sum of the absolute *t*‐values within the cluster. Significance was assessed by comparing the observed cluster statistic against a null distribution generated by randomly permuting condition labels (1000 permutations). The cluster‐level significance threshold was set at alpha = 0.05 (two‐tailed, analysis code is available on OSF: https://osf.io/75mzh/overview).

### 2.8 Source Localization

To determine the anatomical origins of the scalp‐recorded activities and assign them to specific cortical networks, we reconstructed brain activity in source space. To address the inverse problem, we reconstructed brain activity at multiple locations from a limited number of sensors (Pascual‐Marqui 1999). Since individual anatomical data were not available for the participants in this study, the default Brainstorm anatomy was used, and the Boundary Element Method (BEM; Gramfort et al. 2010; Kybic et al. 2005) was applied to compute the volume conduction model of the head.

The source estimation approach employed in this study was minimum‐norm imaging (MN imaging), as implemented in Brainstorm (Baillet et al. 2001). This method estimates the amplitudes of brain sources distributed across the cortical surface by solving the inverse problem under the constraint of minimizing the overall amplitude of brain activity. Noise statistics were obtained from a noise covariance matrix computed using the 2 min resting‐state EEG measurements before movie onset. The noise covariance matrix was regularized by adding a diagonal matrix scaled to 10% of the largest eigenvalue, with a regularization factor of 0.1. In addition, a regularization parameter controls the balance between the signal and noise models in source estimation, with higher SNR values giving more weight to the signal. In this study, an SNR of 3 was used, meaning the signal power was assumed to be nine times greater than the noise power, guiding the inverse solution to prioritize signal components over noise. Subsequently, final source estimates were computed to best fit the sensor‐level data while adhering to the constraints imposed by the cortical surface model and noise covariance (Engemann et al. 2015; Tadel et al. 2019). This principle of adding a scaled identity matrix to stabilize the covariance is consistent with the approaches discussed in the MNE and Brainstorm documentation.

To account for the inhomogeneous sensitivity of EEG to depth and current flow orientation, the current density map was standardized using dynamical Statistical Parametric Mapping (dSPM; Dale et al. 2000). This approach scaled the current density maps relative to the noise covariance, yielding z‐scores. Given that an MRI template was used instead of individual anatomies, a loose orientation model with three dipoles per source grid location (vertex) was employed. This model accounted for anatomical and physiological uncertainties, with the amplitudes of the two additional dipoles restricted to 20% of the main dipole's amplitude. The resulting kernel had dimensions of 3 × NbVertices × NbChannels.

Source calculations were performed using a whole‐brain parcellation approach. The entire cortex was subdivided into 68 anatomical regions based on the Desikan‐Killiany atlas (Desikan et al. 2006), as implemented in FreeSurfer and available in Brainstorm. For each anatomical parcel, the brain activity time series was calculated by averaging the source activity across all vertices within that specific region. This parcel‐based approach was chosen to increase the signal‐to‐noise ratio, reduce the dimensionality of the data for statistical testing and account for spatial blurring inherent to EEG inverse solutions. Because individual anatomical variability was not accounted for due to the use of a template brain, vertex‐level precision is limited; therefore, analysing the data at the parcel level provides a more robust estimate of regional activity. Anatomical terms referenced in this context serve as general indicators derived from this whole‐brain parcellation and should be interpreted as approximate.

### 2.9 EEG Power Extraction and Correlation With Arousal Measures

To directly test the hypothesis that slow‐wave cortical activity tracks the trajectory of emotional arousal, we computed the time‐resolved correlation between EEG power and our arousal measures (arousal annotations and pupil diameter). We focused specifically on Delta, Theta and low‐IAF bands given their hypothesized roles in motivational processing, emotional regulation and sensory gating, respectively.

The PSD was estimated using a 1‐s sliding window with no overlap (1200 epochs total). For each 1‐s epoch, we calculated the PSD using Welch's method (pwelch; Hann window, 200 samples) on the detrended data. We then extracted the mean power within the specific frequency bands of interest: Delta (1–4 Hz), Theta (4–7 Hz), and Alpha (low IAF—IAF). To ensure the EEG power time series were statistically suitable for linear covariance analysis, we applied a three‐step transformation pipeline similar to the pupil preprocessing: (1) Log‐Transformation: Mean power values were log‐transformed (log_10_) to normalize the data distribution and mitigate the influence of outliers (e.g., movement artefacts). (2) Smoothing: To recover the underlying slow‐moving arousal trend and match the temporal dynamics of the autonomic measures, we applied a Gaussian smoothing kernel (window size = 40 s) to the log‐transformed power time series. (3) Standardization (Z‐scoring): Finally, the smoothed time series were z‐scored (mean subtracted, divided by standard deviation). This step effectively removed individual baseline differences and tonic offsets, isolating the phasic relative changes in power over time. The temporal relationship between brain activity and arousal was quantified by calculating the covariance between the processed EEG power time series and the arousal vectors (arousal annotations and pupil diameter). Specifically, the log‐transformed power time series were z‐scored (standardized) at each electrode. This process subtracts the mean across epochs to isolate phasic changes in spectral amplitude while normalizing the variance, similar to other studies using dynamic naturalistic scenarios (Koenig et al. 2008; Mikutta et al. 2012). The arousal vectors were similarly z‐scored. The covariance was then computed as the dot product of the standardized EEG vector and the standardized arousal vector, normalized by the number of time points (N‐1). Because both the EEG and arousal data were z‐scored, this calculation is mathematically equivalent to the Pearson correlation coefficient (analysis code is available on OSF: https://osf.io/75mzh/overview).

### 2.10 Statistical Analysis

To determine whether these correlation values were significantly different from zero across the group, we performed a nonparametric, cluster‐based permutation test. This procedure was implemented using a custom MATLAB script adapted from the spatial clustering and maximum‐statistic framework of the Mass Univariate ERP Toolbox (Groppe et al. 2011a). This approach effectively accounts for the spatial dependence of neighbouring electrodes and provides robust control for the family‐wise error rate (FWER) across multiple comparisons (Groppe et al. 2011a, 2011b; Maris and Oostenveld 2007; Pernet et al. 2015). The input dataset consisted of a spatial topography of correlation values for each participant (channels × subjects). First, a one‐sample *t*‐test was conducted at each channel to test whether the mean correlation differed significantly from zero. An uncorrected, two‐tailed significance threshold (alpha = 0.05) was applied to these empirical *t*‐values. To identify spatial clusters, the neighbourhood structure of the EEG cap was defined using a spatial adjacency matrix derived from standard electrode positions, with a distance threshold of 0.4. Clusters were identified using a custom spatial‐connectivity algorithm: adjacent channels exceeding the uncorrected *t*‐threshold were grouped into a continuous cluster only if they shared the same sign (positive or negative). The cluster‐level statistic (cluster mass) was then calculated by summing the absolute *t*‐values of all electrodes within a given cluster. To assess the statistical significance of these empirical clusters, we generated a null distribution using 10,000 permutations. In each permutation, the signs of the correlation values were randomly flipped for each participant, and new *t*‐values were computed across all channels. Spatial clusters were formed in this permuted data using the exact same adjacency rules, and only the maximum cluster mass from each permutation was retained to build the null distribution. Finally, a corrected *p*‐value was calculated for each empirical cluster by determining the proportion of the null distribution that exceeded the empirical cluster mass. Clusters with a corrected *p* < 0.05 were considered statistically significant (analysis code is available on OSF: https://osf.io/75mzh/overview).

Additionally, Pearson's correlation was used to assess the relationship between the group‐level average trajectories of the subjective arousal annotations and pupil diameter.

## 3 Results

### 3.1 Stimulus Validation: 1/f and ISC

**FIGURE 1:**
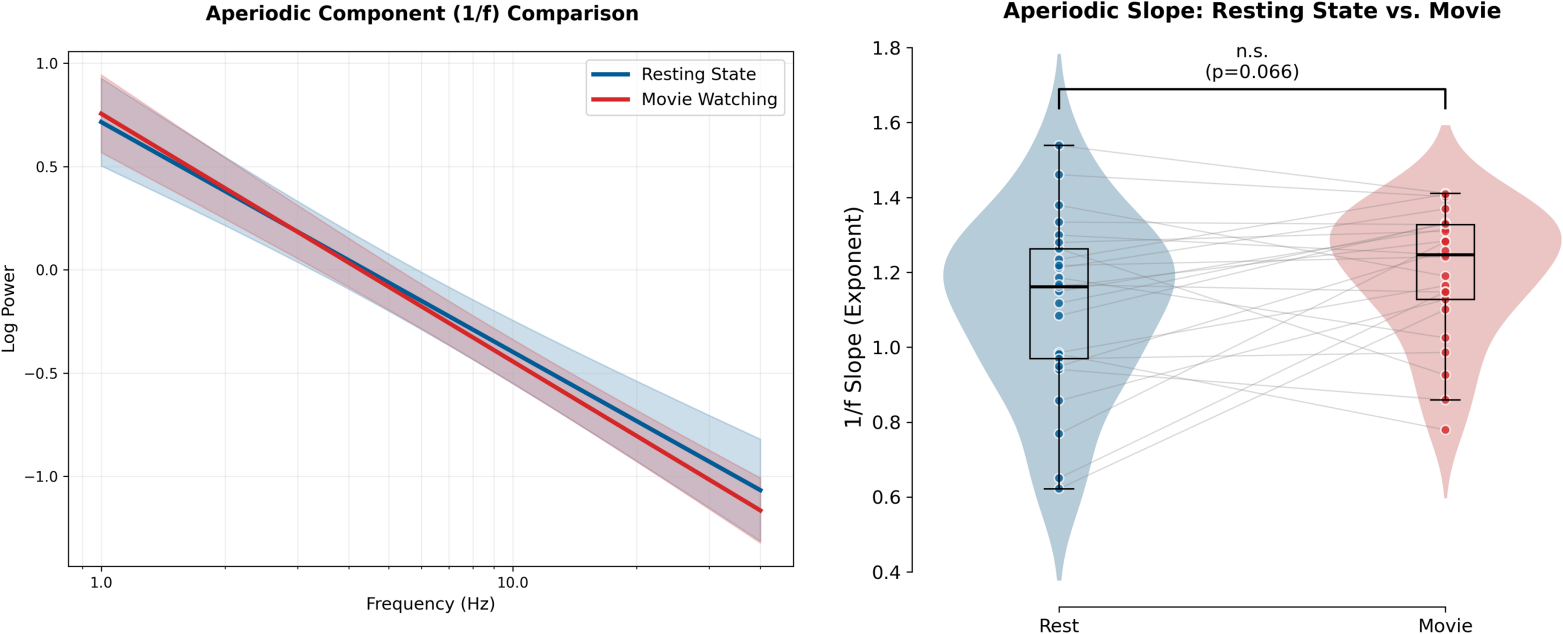
Comparison of the global aperiodic slope (1/f) between resting state and movie viewing. The figure illustrates the differences in the global aperiodic component of the power spectrum between the Resting State (blue) and Movie Viewing (red) conditions. (A) The left panel displays the grand average global aperiodic slopes in log–log space. The x‐axis represents log‐transformed frequency, and the y‐axis represents log‐transformed power. The steeper slope observed in the movie condition indicates a shift in the broadband 1/f activity across the scalp. (B) The right panel shows raincloud plots comparing the distribution of global aperiodic exponents (1/f slope) for resting state (blue) and movie viewing (red). Individual data points represent single participants (*N* = 25), with boxplots indicating the median and interquartile range. The difference between conditions was marginally nonsignificant (*p* = 0.066, paired *t*‐test), with the movie viewing condition showing a trend toward a higher (steeper) exponent compared to resting state.

**FIGURE 2:**
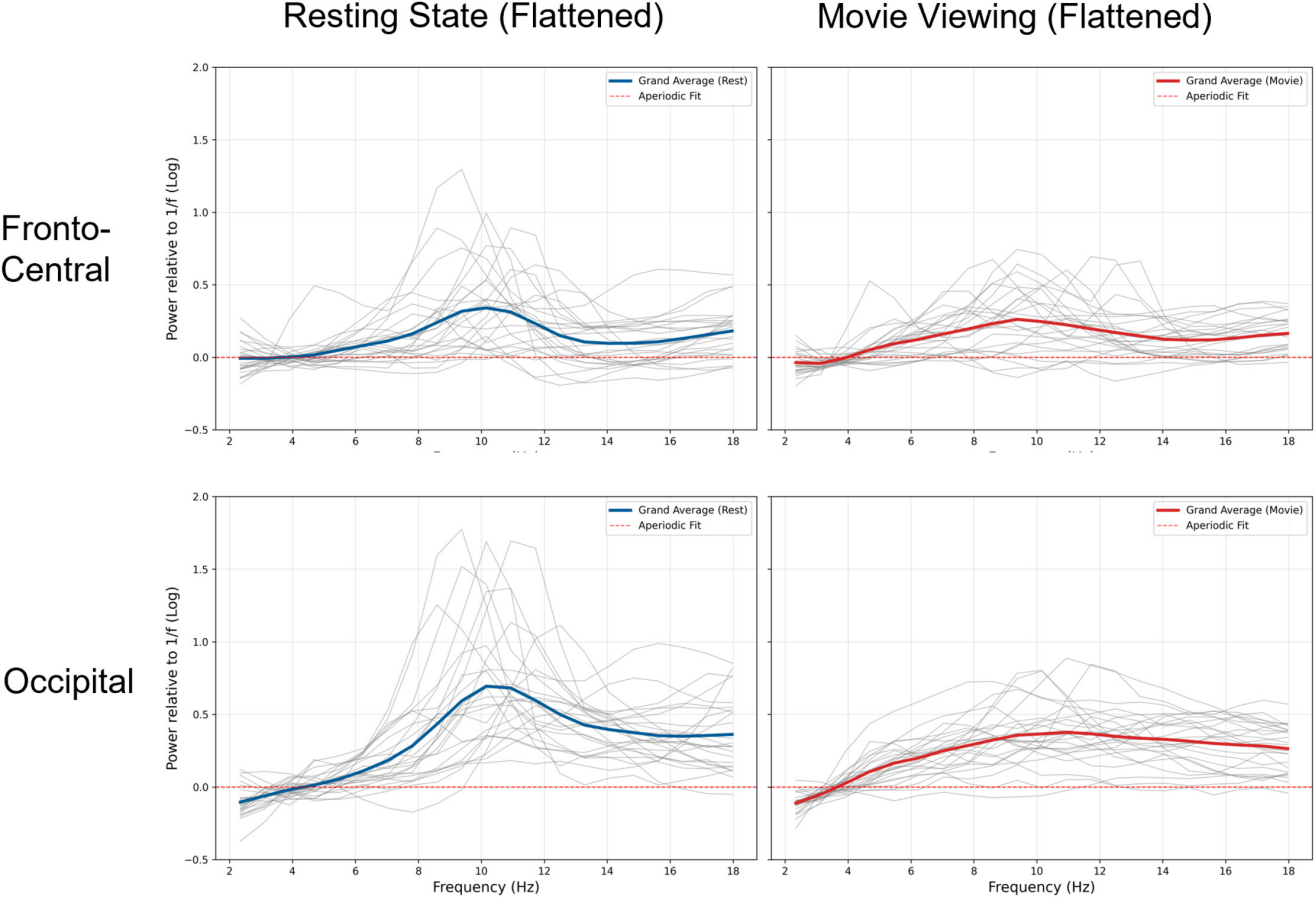
Flattened power spectra comparing resting state and movie viewing across fronto‐central and occipital regions. The figure displays the aperiodic‐corrected (flattened) power spectra derived using the SpecParam algorithm. The top row illustrates the Fronto‐Central region, while the bottom row illustrates the Occipital region. In both rows, the left panel represents the 2‐min Resting State condition, and the right panel represents the first 2 min of Movie Viewing. The y‐axis represents log‐transformed power relative to the 1/f aperiodic component, while the x‐axis displays frequency (Hz). The horizontal red dashed line at 0 indicates the fitted aperiodic background; values above this line represent oscillatory peaks (periodic activity) exceeding the 1/f noise floor. Thin grey lines depict the spectra of individual participants (*N* = 25). The thick solid lines represent the group grand average, coloured blue for the Resting State condition and red for the Movie Viewing condition.

**FIGURE 3:**
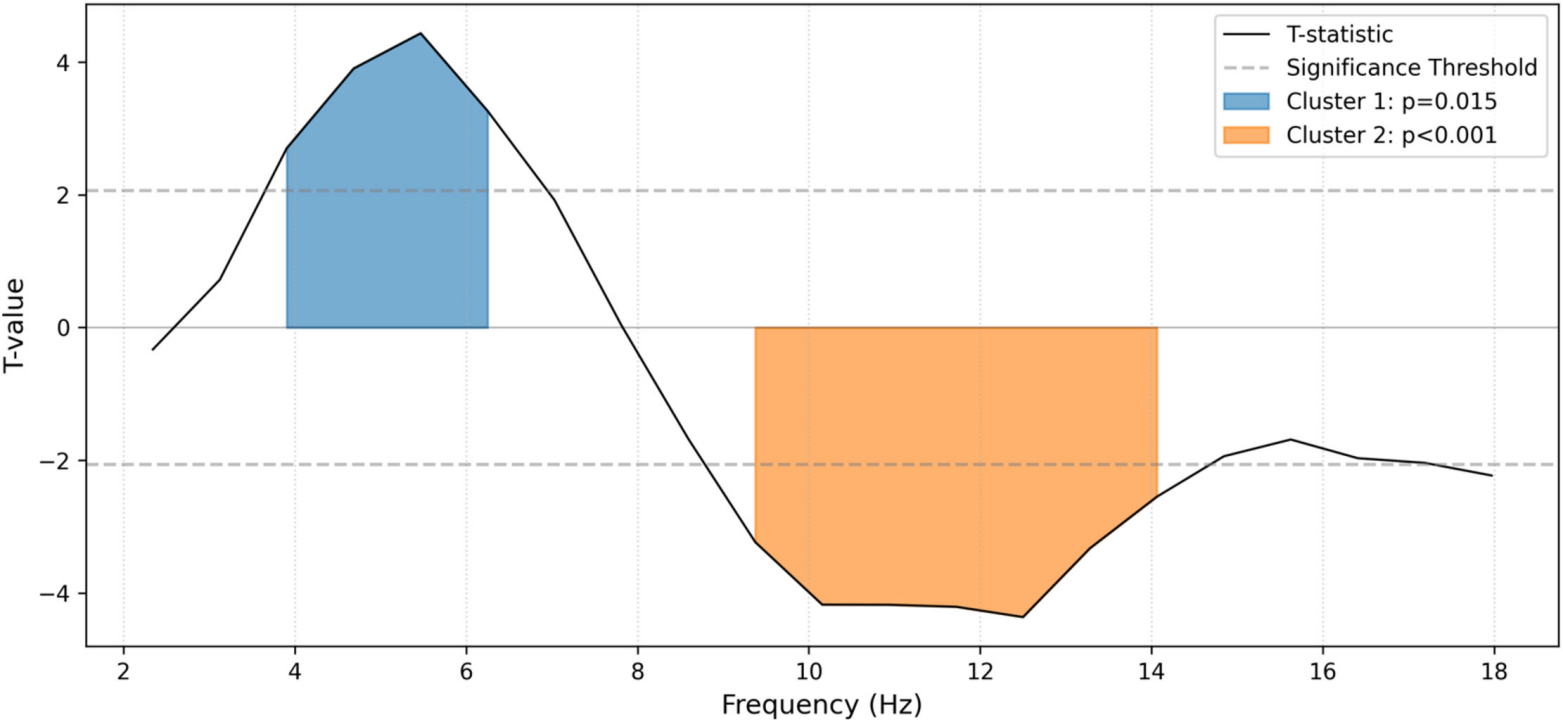
Cluster‐based permutation test results comparing occipital power spectra between movie viewing and resting state. The figure displays the t‐statistics (black solid line) for the comparison of flattened power spectra between the Movie Viewing and Resting State conditions across the frequency range of 2–18 Hz in the Occipital region. The y‐axis represents the *t*‐value, and the x‐axis represents frequency (Hz). The horizontal dotted grey lines at approx 2 indicate the uncorrected significance threshold (alpha = 0.05). Significant frequency clusters identified by the nonparametric permutation test are highlighted in colour: the blue shaded region in the theta frequency range indicates a cluster of significantly higher power during movie viewing (positive *t*‐values), while the yellow shaded region in the alpha frequency range indicates a cluster of significantly lower power during movie viewing (negative *t*‐values).

To verify that the movie induced a global shift in brain state, we analysed the aperiodic (1/f) component. We observed a nonsignificant trend toward a steeper spectral slope during movie watching compared to rest (*t*(24) = −1.93, *p* = 0.066, see Figure 1). Beyond the aperiodic changes, the periodic activity was further characterized, specifically, the spectral differences between conditions (movie vs. rest). To this end, we compared the flattened (aperiodic‐removed) power spectra using cluster‐based permutation testing. In the occipital region (Figures 2 and 3), movie watching was characterized by a significant decrease in alpha power range (approx. 9–14 Hz; *t*_sum_ = −26.03, *p* < 0.001) and a significant increase in theta power range (approx. 4–7 Hz; *t*_sum_ = 14.30, *p* = 0.015) compared to resting state. Parallel analyses conducted on fronto‐central channels revealed no significant clusters (see Figure 2), suggesting that these tonic oscillatory shifts were topographically specific to posterior sensory regions. This regional modulation confirms that the movie stimulus successfully engaged brain activity, providing a physiological basis for our subsequent correlation analyses.

**FIGURE 4:**
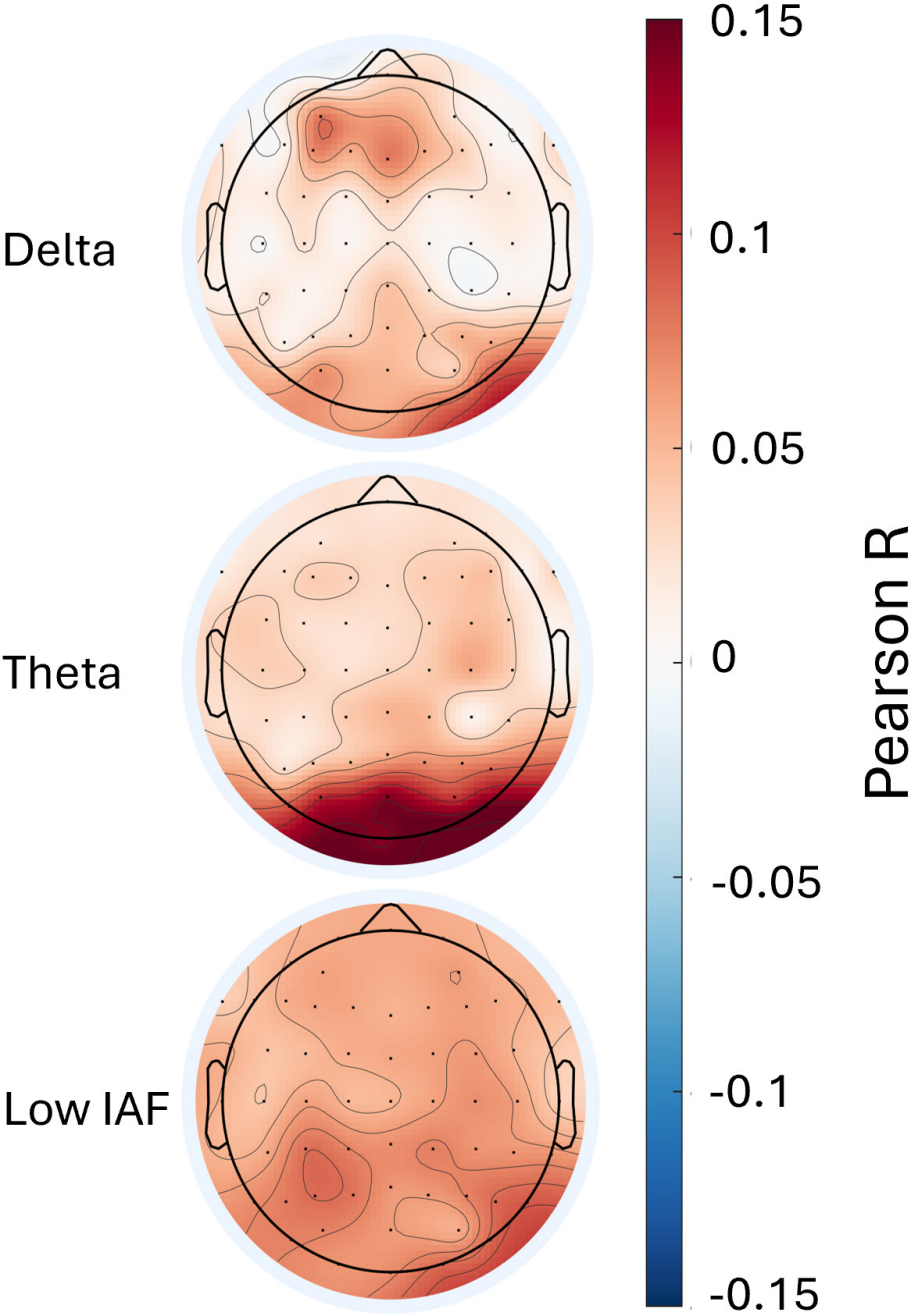
Inter‐subject correlation of neural oscillations across frequency bands during movie viewing. Topographical maps show ISC for delta (2–4 Hz), theta (4–7 Hz) and individual low alpha frequency power. The colour bar indicates Pearson's r. The theta band exhibits the strongest occipital synchronization, consistent with prior findings on movie viewing paradigms. *ISC*, Inter‐subject correlation.

The ISC analysis revealed robust alignment across the cohort for both the time series of arousal annotations (*n* = 35) and pupil diameter (*n* = 30). The average ISC was r = 0.51 ± 0.27 (*p* < 0.001) for arousal annotations (*n* = 35) and *r* = 0.66 ± 0.44 (*p* < 0.001) for pupil diameter (*n* = 30). All statistical tests were performed on Fisher z‐transformed correlation coefficients. In contrast to the highly synchronized behavioural and pupillary responses, the raw ISC of EEG spectral power across the scalp was generally lower in magnitude. To descriptively characterize the spatial distribution of these values without formal statistical thresholding, topographical mapping (Figure 4) was used to observe spatial trends across the delta (2–4 Hz), theta (4–7 Hz) and low individual alpha (low IAF) bands. Qualitatively, the highest neural synchronization values were observed over occipital regions, particularly in the theta band (maximum uncorrected *r* = 0.17). The delta band exhibited localized peaks of synchronization in both occipital and left‐frontal regions (maximum uncorrected *r* = 0.092). Finally, the low IAF band displayed a similar occipital topography to theta, albeit with lower overall ISC magnitudes (maximum uncorrected *r* = 0.092).

**FIGURE 5:**
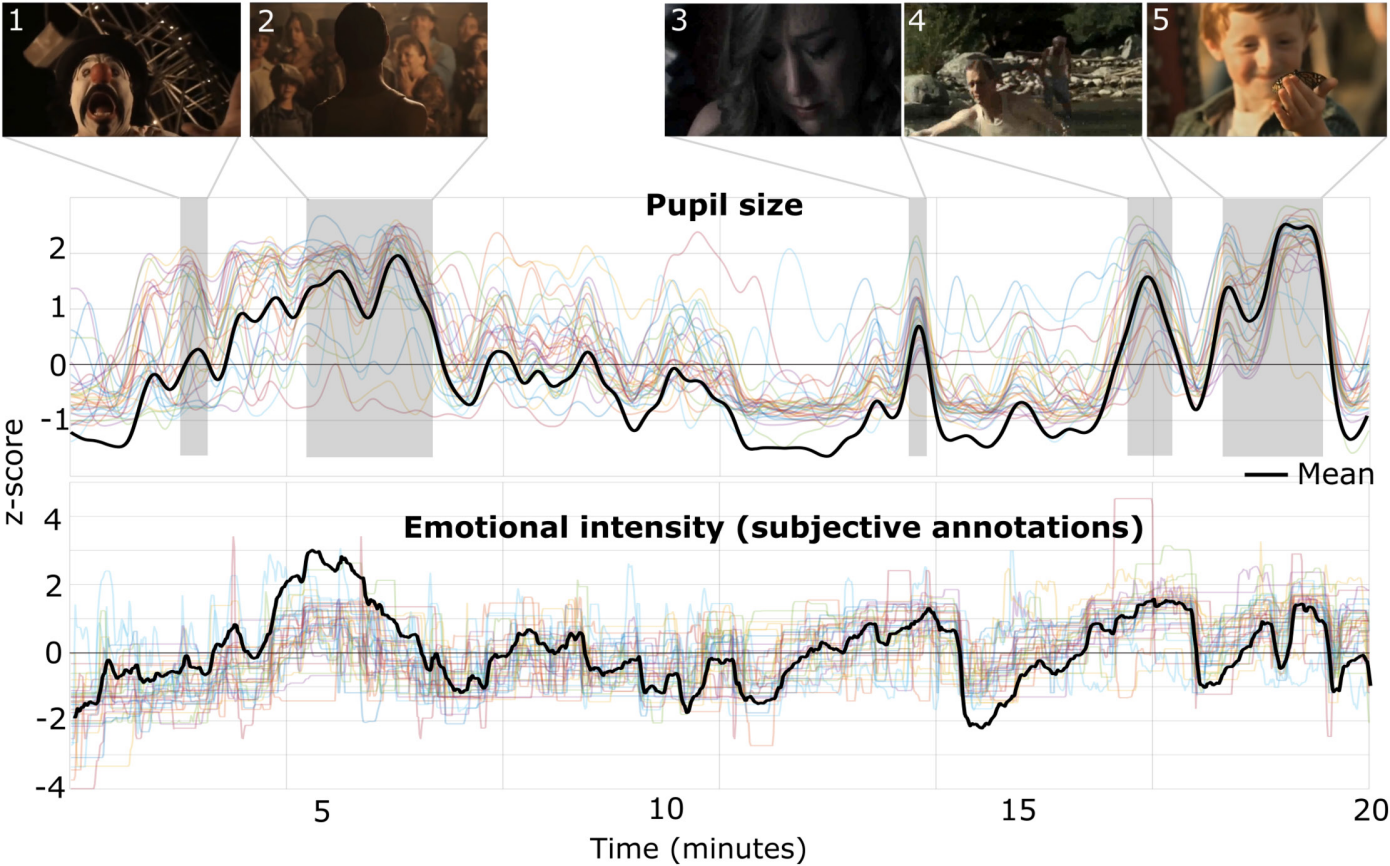
Objective and subjective arousal annotations during viewing of the short movie Butterfly Circus. The upper panel depicts pupil size throughout the entire duration of the movie (20 min). The lower panel presents retroactive subjective arousal annotations. Each coloured line represents the individual participant timeseries, while the black line indicates the mean. Peaks higher than 0 (z‐score) matching both modalities are highlighted in grey with the corresponding scene of the movie. Scene's description, 1: Clown appearance with loud laughter and exaggerated sounds. 2: Will (protagonist) is presented dramatically. 3: A woman is shown crying after experiencing mistreatment. 4: Will is submerged in the water as others search for him with concern. 5: A child releases a butterfly as surrounding characters smile, conveying a celebratory ending.

We further found that time‐resolved patterns of arousal annotations and pupil diameter are correlated. A Pearson correlation analysis revealed that the mean of arousal annotations was correlated with the mean of pupil diameter (*r* = 0.404, *p* < 0.001), indicating a shared variance between subjective experiences and physiological responses (see Figure 5).

### 3.2 Rationale for Multi‐Level Analysis: Bridging Shared Behaviour and Individual Neural Dynamics

Our analysis revealed a clear divergence between behavioural and neural synchronization. While arousal annotations and pupil diameter showed high ISC, confirming that the movie successfully elicited shared emotional trajectories (Dmochowski et al. 2012; Hofmann et al. 2021; Nanni‐Zepeda et al. 2024), the raw EEG oscillatory power showed lower group‐level synchronization. To isolate the shared arousal signal from these individualized neural differences, we used the average of the arousal annotations and pupil diameter measures. By correlating each participant's moment‐to‐moment power with the group‐level arousal dynamics, we used the robust, shared behavioural signal as a ‘functional localizer’ to identify systematic neural modulations that might otherwise be masked in a purely group‐averaged neural analysis. This approach allows us to detect shared neuro‐oscillatory mechanisms tracking arousal (see Figure 4).

### 3.3 Arousal Annotations and Pupil Diameter Share Oscillatory Signatures

**FIGURE 6:**
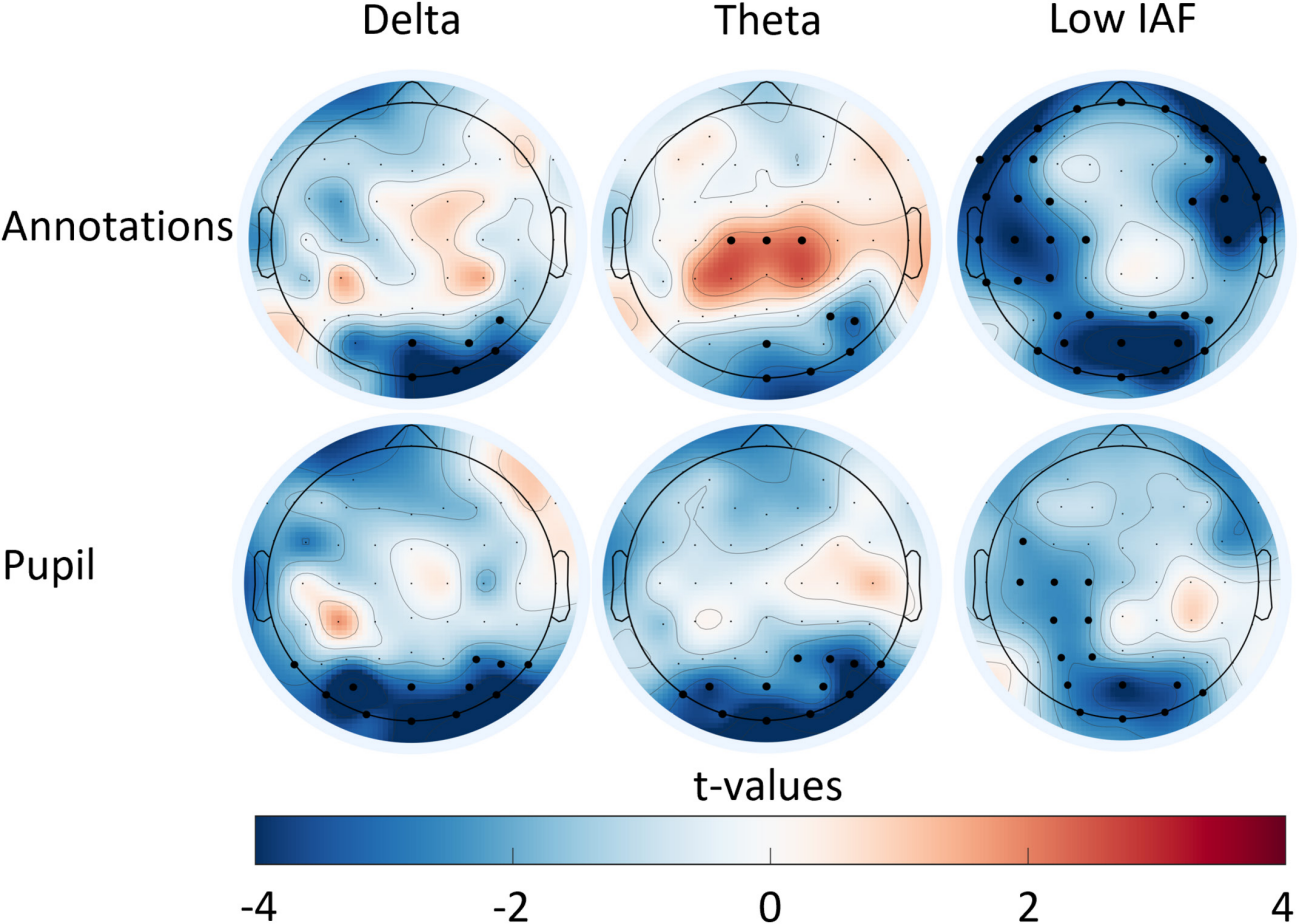
Correlation of oscillations with arousal across frequency bands. Topographical maps depict significant clusters of channels showing correlation between power time series and the average over the arousal annotation time series (top row) as well as pupil dilation time series (bottom row) for three frequency bands: delta (2–4 Hz), theta (4–8 Hz) and individual low alpha. The colour bar represents *t*‐values, ranging from −4 (blue) to 4 (red). Black dots indicate channels forming significant clusters (*p* < 0.05).

**FIGURE 7:**
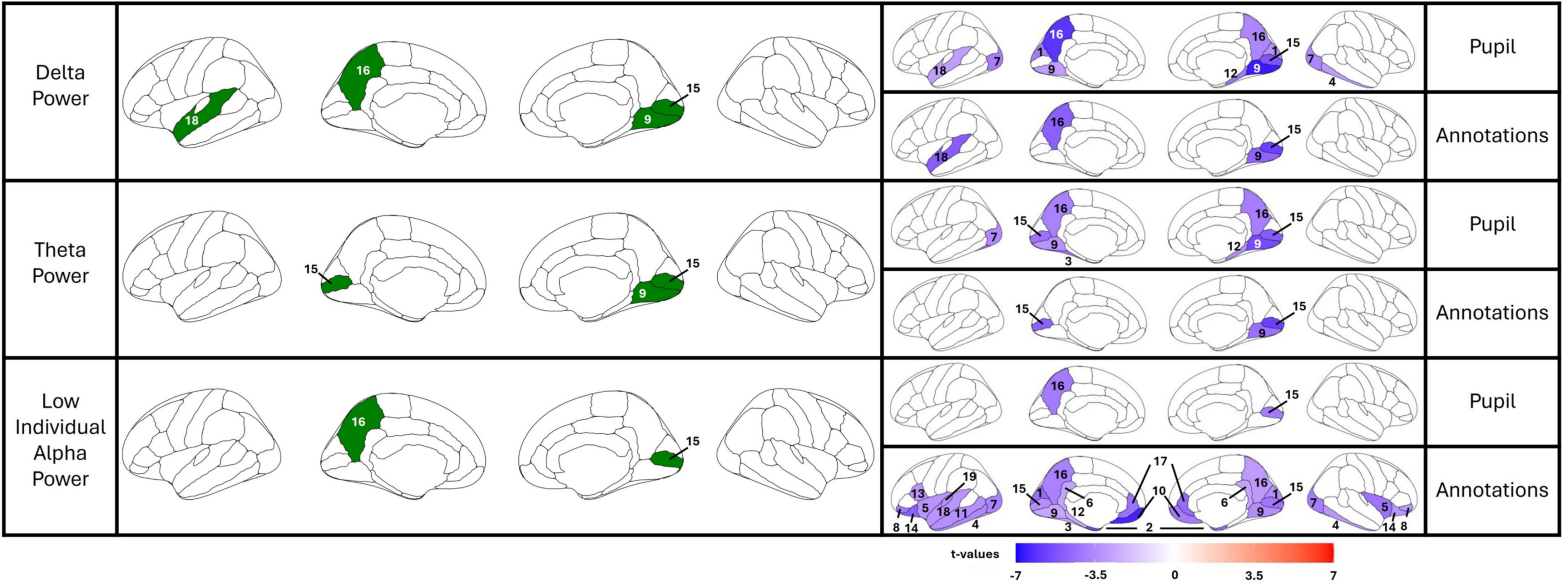
Brain sources significantly associated with mean arousal annotations and mean pupil diameter for delta, theta and low Individual Alpha Frequency (IAF) power. The figure illustrates the brain sources whose power significantly correlated with mean arousal annotations and pupil diameter in the delta (upper panel), theta (middle panel) and low IAF frequency bands. The left column highlights sources (indicated in green) where power correlated significantly with both arousal annotations and pupil diameter. The right column is divided into two sections: one showing sources where power correlated significantly with mean pupil diameter, and the other showing sources where power correlated significantly with mean arousal annotations. The colour bar at the bottom represents *t*‐values (range: −7 to 7, indicated in blue‐to‐red colour), indicating the results of a one‐sample *t*‐test assessing if the correlation coefficients differed from zero. Cooler colours indicate regions where the correlation values were significantly smaller than zero. All presented results are significant after FDR correction (pFDR < 0.05). Regions: (1) cuneus, (2) entorhinal, (3) fusiform, (4) inferiortemporal, (5) insula, (6) isthmuscingulate, (7) lateraloccipial, (8) lateralorbitofrontal, (9) lingual, (10) medialorbitofrontal, (11) middletemporal, (12) parahippocampal, (13) parsopercularis, (14) parsorbitalis, (15) pericalcarine, (16) precuneus, (17) rostralanteriorcingulate, (18) superiortemporal, (19) transversetemporal.

To identify oscillatory signatures underlying both arousal annotations and pupil diameter, we focused on their shared patterns across sensor and source space (Figures 6 and 7). All *p*‐values reported for source space analyses were FDR‐corrected, while sensor space results were corrected using cluster‐based permutation tests. Crucially, for sensor‐level inference, we report the ‘Cluster Mass’ (the sum of *t*‐values across all channels in the cluster) to assess significance against the permutation distribution, while also reporting the ‘Peak *t*’ (the maximum t‐statistic at a single channel) and Cohen's *d* to characterize the local effect strength.

#### 3.3.1 Sensor Space Results

As detailed in Table 1, distinct oscillatory patterns emerged across frequency bands. In the Delta (2–4 Hz), Theta (4–7 Hz) and low IAF bands, we observed significant clusters of correlation that were largely consistent across both arousal annotations and pupil diameter measures. Specifically, in the Low‐IAF range, we observed widespread negative clusters across (fronto‐)parieto‐occipital regions for both arousal annotations (Cluster Mass = −131.55, peak *t*(24) = −5.62, *p* < 0.001) and pupil diameter (Cluster Mass = −39.59, peak *t*(24) = −4.03, *p* < 0.001). For the delta and theta bands, significant negative clusters were located primarily in the occipital regions for both annotations (Delta: Cluster Mass = −20.60, peak *t*(24) = −4.47, *p* = 0.012; Theta: Cluster Mass = −16.57, peak *t*(24) = −3.28, *p* < 0.001) and pupil diameter (Delta: Cluster Mass = −45.57, peak t(24) = −6.03, *p* < 0.001; Theta: Cluster Mass = −42.43, peak *t*(24) = −5.48, *p* = 0.012). Notably, we also observed a unique, significant positive correlation cluster in the theta band over central regions, which was specific to subjective arousal annotations (Cluster Mass = 6.40, peak *t*(24) = 2.24, *p* = 0.009).

**TABLE 1:** Significant sensor‐level clusters correlating with arousal annotations and pupil diameter.

Frequency band	Arousal measure	Region	No. channels	Cluster mass (*t*_sum_)	*p*	Peak *t*(24)	Effect size (Cohen's *d*)	Avg. correlation (*r*)
Delta	Annotations	Right occipital	6	−20.6	0.012	−4.47	−0.89	−0.12
	Pupil diameter	Occipital	12	−45.57	< 0.001	−6.03	−1.21	−0.08
Theta	Annotations	Occipital	6	−16.57	< 0.001	−3.28	−0.66	−0.09
		Central	3	6.4	0.009	2.24	0.45	0.08
	Pupil diameter	Occipital	12	−42.43	0.012	−5.48	−1.1	−0.12
Low IAF	Annotations	Fronto‐parieto‐occipital	39	−131.55	< 0.001	−5.62	−1.12	−0.09
	Pupil diameter	Parieto‐occipital	15	−39.59	< 0.001	−4.03	−0.81	−0.09

*Note:* Cluster Mass represents the sum of *t*‐values across all channels within the significant cluster. Peak *t*_(24)_ denotes the maximum t‐statistic observed at a single channel within the cluster. Effect Size *d* is Cohen's *d*, calculated from the peak *t*‐statistic (*d* = *t*/sqrt(*N*)). Avg. Correlation (*r*) represents the mean Pearson correlation coefficient averaged across all channels and participants within the cluster.

Abbreviation: IAF = individual alpha frequency.

#### 3.3.2 Source Space Results

Source localization (detailed in Table 2) confirmed that these scalp topographies originated from a distributed network of cortical generators, with specific regions demonstrating spatial convergence between both arousal measures across frequency bands. In the delta band, overlapping significant correlation was localized to the right pericalcarine (annotations: *t*(24) = −5.67, *p* (FDR) = 0.001; pupil: *t*(24) = −5.16, *p* (FDR) = 0.001), left precuneus (annotations: *t*(24) = −4.80, *p* (FDR) = 0.002; pupil: *t*(24) = −6.05, *p* (FDR) < 0.001), right lingual gyrus (annotations: *t*(24) = −4.67, *p* (FDR) = 0.002; pupil: *t*(24) = −6.62, *p* (FDR) < 0.001) and left superior temporal gyrus (STG) (annotations: *t*(24) = −4.92, *p* (FDR) = 0.002; pupil: *t*(24) = −2.92, *p* (FDR) = 0.045). In the theta band, overlapping significant regions included the right pericalcarine (annotations: *t*(24) = −5.71, *p* (FDR) < 0.001; pupil: *t*(24) = −4.93, *p* (FDR) = 0.002), left pericalcarine (annotations: *t*(24) = −4.72, *p* (FDR) = 0.003; pupil: *t*(24) = −4.24, *p* (FDR) = 0.007) and right lingual gyrus (annotations: *t*(24) = −4.29, *p* (FDR) = 0.006; pupil: *t*(24) = −5.40, *p* (FDR) = 0.001). Finally, in the low IAF band, shared neural substrates were found in the left precuneus (annotations: *t*(24) = −3.79, *p* (FDR) < 0.001; pupil: *t*(24) = −4.00, *p* (FDR) = 0.018) and right pericalcarine (annotations: *t*(24) = −4.16, *p* (FDR) < 0.001; pupil: *t*(24) = −3.99, *p* (FDR) = 0.018). As detailed in Table 2, the effect sizes across these overlapping regions were substantial (Cohen's d ranging from −0.53 to −1.32), indicating a robust link between cortical oscillations and the shared trajectory of emotional arousal.

**TABLE 2:** Source‐level anatomical parcels correlating with arousal measures.

Frequency band	Arousal measure	Region	*t* (24)	*p* (FDR)	Effect size (Cohen's *d*)	Avg. correlation (*r*)
Delta	Annotations	Lingual R	−4.67	0.002	−0.93	−0.11
		Pericalcarine R	−5.67	0.001	−1.13	−0.15
		Precuneus L	−4.8	0.002	−0.96	−0.12
		Superiortemporal L	−4.92	0.002	−0.98	−0.14
	Pupil diameter	Cuneus L	−3.88	0.008	−0.78	−0.11
		Cuneus R	−3.38	0.019	−0.68	−0.09
		Inferiortemporal L	−2.93	0.045	−0.59	−0.067
		Lateraloccipital L	−3.86	0.008	−0.77	−0.14
		Lateraloccipital R	−3.62	0.012	−0.72	−0.09
		Lingual L	−2.89	0.045	−0.58	−0.08
		Lingual R	−6.62	< 0.001	−1.32	−0.15
		Parahippocampal R	−4.06	0.008	−0.81	−0.11
		Pericalcarine R	−5.16	0.001	−1.03	−0.13
		Precuneus L	−6.05	< 0.001	−1.21	−0.15
		Precuneus R	−3.64	0.012	−0.73	−0.13
		Superiortemporal L	−2.92	0.045	−0.58	−0.08
Theta	Annotations	Lingual R	−4.29	0.006	−0.86	−0.13
		Pericalcarine L	−4.72	0.003	−0.94	−0.14
		Pericalcarine R	−5.71	< 0.001	−1.14	−0.18
	Pupil diameter	Fusiform L	−4.12	0.007	−0.82	−0.12
		Lateraloccipital L	−3.50	0.018	−0.70	−0.13
		Lingual L	−3.22	0.028	−0.64	−0.1
		Lingual R	−5.40	0.001	−1.08	−0.15
		Parahippocampal R	−3.34	0.023	−0.67	−0.11
		Pericalcarine L	−4.24	0.007	−0.85	−0.14
		Pericalcarine R	−4.93	0.002	−0.99	−0.16
		Precuneus L	−3.78	0.010	−0.76	−0.12
		Precuneus R	−3.88	0.010	−0.78	−0.11
Low IAF	Annotations	Cuneus L	−4.40	< 0.001	−0.88	−0.13
		Cuneus R	−3.35	0.01	−0.67	−0.10
		Entorhinal L	−5.36	< 0.001	−1.07	−0.16
		Entorhinal R	−4.42	< 0.001	−0.88	−0.13
		Frontalpole L	−3.04	< 0.01	−0.61	−0.09
		Fusiform L	−2.53	< 0.04	−0.51	−0.08
		Inferiortemporal L	−3.60	< 0.01	−0.72	−0.10
		Inferiortemporal R	−3.13	< 0.01	−0.63	−0.11
		Insula L	−3.21	< 0.01	−0.64	−0.09


		Insula R	−4.20	< 0.001	−0.84	−0.09
		Isthmuscingulate L	−2.74	0.02	−0.55	−0.06
		Isthmuscingulate R	−2.73	0.02	−0.55	−0.07
		Lateraloccipital L	−3.76	< 0.001	−0.75	−0.10
		Lateraloccipital R	−3.91	< 0.001	−0.78	−0.14
		Lateralorbitofrontal L	−4.80	< 0.001	−0.96	−0.12
		Lateralorbitofrontal R	−3.17	0.01	−0.63	−0.09
		Lingual L	−2.64	0.03	−0.53	−0.10
		Lingual R	−3.41	0.01	−0.68	−0.13
		Medialorbitofrontal L	−6.43	< 0.001	−1.29	−0.13
		Medialorbitofrontal R	−3.93	< 0.001	−0.79	−0.11
		Middletemporal L	−3.32	0.01	−0.66	−0.09
		Parahippocampal L	−2.46	0.04	−0.49	−0.08
		Parsopercularis L	−3.23	0.01	−0.65	−0.10
		Parsorbitalis L	−4.06	< 0.001	−0.81	−0.13
		Parsorbitalis R	−3.14	0.01	−0.63	−0.10
		Pericalcarine L	−3.31	0.01	−0.66	−0.13
		Pericalcarine R	−4.16	< 0.001	−0.83	−0.16
		Precuneus L	−3.79	< 0.001	−0.76	−0.11
		Precuneus R	−3.01	0.01	−0.60	−0.07
		Rostralanteriorcingulate L	−4.24	< 0.001	−0.85	−0.11
		Rostralanteriorcingulate R	−4.49	< 0.001	−0.90	−0.12
		Superiortemporal L	−3.17	0.01	−0.63	−0.10
		Temporalpole L	−4.87	< 0.001	−0.97	−0.16
		Temporalpole R	−3.98	< 0.001	−0.80	−0.12
	Transversetemporal L	−3.28	0.01	−0.66	−0.11
Pupil diameter	Pericalcarine R	−3.99	0.018	−0.80	−0.15
	Precuneus L	−4.00	0.018	−0.80	−0.12

*Note:* Results are based on one‐sample *t*‐tests of the correlation values against zero. p_FDR_ indicates *p*‐values corrected for multiple comparisons using the False Discovery Rate method. Effect Size Cohen's *d* is calculated as t/sqrt(N). Avg. Correlation (r) represents the mean correlation coefficient averaged across all participants within the anatomical parcel.

Abbreviations: L = left hemisphere, R = right hemisphere.

## 4 Discussion

Our study identified neural oscillatory correlates of continuous moment‐to‐moment changes in both annotations and pupil markers of emotional arousal during movie viewing. Before dissecting these specific neural signatures, we first established that the naturalistic stimulus elicited reliable brain states distinct from rest, marked by a steepening of the aperiodic slope and significant modulation of occipital narrowband power (Figures 1, 2, 3). Furthermore, robust ISC in the arousal annotations and pupil diameter time courses confirmed that the film successfully induced synchronized emotional responses across the cohort. With this physiological validity established, we calculated the average across individual timeseries to extract dominant, shared trajectories for both subjective arousal and objective pupil diameter. By correlating these shared group‐level arousal signals with individual brain oscillations, we observed widespread, highly consistent negative correlations across the delta, theta and low‐frequency alpha bands, localized primarily to shared posterior cortical networks (e.g., precuneus, pericalcarine and lingual gyrus). In addition to these shared posterior dynamics, we observed spatial topographies specific to the subjective annotations; aligning with prior hypotheses regarding emotional activation (Mikutta et al. 2012), subjective arousal was associated with low‐frequency alpha desynchronization over right‐frontal regions, as well as unique positive central theta synchronization. This positive central theta cluster stands in direct contrast to the posterior theta desynchronization shared with pupil diameter, highlighting divergent spatial topographies within the theta band. This cross‐level mapping emphasizes stimulus‐locked, shared aspects of emotional and attentional processing. In the following sections, we dissect the specific roles of these distinct brain regions and low‐frequency rhythms (delta, theta and low IAF) in orchestrating the physiological and subjective components of the narrative emotional experience.

### 4.1 Bottom‐Up Control of Visual Processing

In the pericalcarine and lingual gyri, we observed a robust negative correlation between arousal measures (both pupil diameter and arousal annotations) and low‐frequency oscillatory power (alpha, theta and delta) (see Figures 6 and 7). This pattern is consistent with the well‐established phenomenon of event‐related desynchronization (ERD), where a suppression of low‐frequency rhythms marks a transition from a resting ‘idling’ state to an active processing mode.

The pericalcarine cortex (primary visual cortex, V1) and the lingual gyrus are critical for early visual processing and the encoding of complex visual features (Huff et al. 2025; Rich et al. 2006; Roland and Gulyás 1995). Under conditions of low arousal or sensory disengagement, these neuronal populations typically exhibit high amplitude, synchronized oscillations in the low frequency range (delta, theta, alpha), reflecting a state of functional inhibition or ‘gating’ of sensory input (Foxe and Snyder 2011; Jensen and Mazaheri 2010; Klimesch et al. 2007; G. G. Knyazev 2007). Conversely, the arrival of visually intense or emotionally arousing stimuli requires the rapid processing of high‐dimensional sensory information. This demand triggers a ‘blockade’ of these inhibitory rhythms, resulting in the desynchronized, low‐power state we observed during high‐arousal epochs.

The strong link between this desynchronization and pupil diameter provides specific insight into the neurophysiological mechanism driving this state change. Pupil diameter is a reliable noninvasive proxy for activity in the Locus Coeruleus (LC), the brain's primary source of norepinephrine (Joshi et al. 2016; Murphy et al. 2014). Recent animal models have demonstrated that LC‐norepinephrine (LC‐NE) projections to the visual cortex play a causal role in modulating cortical state. Specifically, phasic activation of the LC (indexed by pupil diameter) suppresses low‐frequency fluctuations (delta/theta/alpha) and shifts the cortex into a desynchronized, high‐conductance state (McGinley et al. 2015; Polack et al. 2013; Vinck et al. 2015). This norepinephrine‐mediated suppression of slow waves serves to enhance the signal‐to‐noise ratio of sensory neurons, thereby sharpening visual encoding during periods of heightened arousal (Mather and Harley 2016).

Crucially, this local periodic desynchronization aligns with the global neurophysiological state we observed during our initial stimulus validation, i.e., the steepening of the aperiodic 1/f slope (Figure 1). While task activation is often associated with a flattened spectrum (Gao et al. 2017), recent work demonstrates that steeper slopes during naturalistic viewing reflect stronger neural inhibition and selective sensory gating (Manyukhina et al. 2024; Tronelli et al. 2025). Thus, as arousal increases, a ‘bottom‐up’ control mechanism emerges: LC‐NE activity suppresses specific periodic low‐frequency rhythms to facilitate visual throughput, while heightened global neural inhibition, reflected by the steepened 1/f slope, simultaneously suppresses asynchronous background noise. Together, this creates a state of highly efficient, inhibition‐supported sensory entrainment required for narrative immersion.

### 4.2 Top‐Down Cognitive‐Emotional Integration

A key finding of this study in the sensor space was the specific association between subjective arousal annotations and theta oscillatory activity over central regions (see Figure 6). While this maximal scalp power emerged centrally, this topography is highly characteristic of the well‐established mid‐frontal theta network. Because of the orientation of underlying cortical generators, specifically within the medial prefrontal and anterior cingulate cortices, mid‐frontal theta signals frequently project to central scalp electrodes (Cavanagh and Frank 2014). To understand this pattern, we must look at the functional role of this fronto‐central network. While posterior regions handle early sensory encoding, frontal midline theta mediates the higher‐order integration of sensory information with internal affective states (Adamczyk and Wyczesany 2023; Hyman et al. 2005; Symons et al. 2016). During naturalistic viewing, evaluating emotional arousal is not a passive reflex; it is an active cognitive process. Generating a subjective arousal annotation requires the viewer to continuously bind incoming narrative data with internal emotional models to judge a scene's affective weight. Therefore, the robust correlation between this central theta signature and arousal annotations likely reflects this top‐down, cognitive‐emotional integration.

This framework also highlights the unique cognitive demands of subjective reporting compared to autonomic tracking. Although pupil size is a well‐established proxy for LC‐Norepinephrine activity and global physiological arousal (Berridge and Waterhouse 2003; Joshi et al. 2016; Mather and Harley 2016; Murphy et al. 2014; Sara and Bouret 2012), its response during movie watching is heavily driven by low‐level sensory characteristics (e.g., visual transients and motion) rather than high‐level semantic meaning. Ultimately, while both measures capture the intensity of the experience, the pronounced central theta tracking observed for subjective annotations specifically reflects the top‐down cognitive elaboration required to evaluate the narrative's emotional content.

### 4.3 Auditory Processing

We observed widespread negative correlations between arousal measures and low‐frequency oscillations (delta and low IAF) localized to the STG. While sensor‐level analysis for low IAF showed significant clusters for both measures, source‐space analysis provided specific insights into how subjective arousal relates to cortical excitability within these regions (see Figures 6 and 7).

Specifically, periods of high arousal annotations were characterized by significant suppression (desynchronization) of low IAF and delta power in the left STG, a hub for auditory processing, speech perception and lexical access (Leff et al. 2009; Yi et al. 2019). In sensory cortices, high‐amplitude alpha and delta typically reflect functional inhibition or ‘idling’, while their suppression serves as a robust marker of cortical activation excitability (Jensen and Mazaheri 2010; Klimesch et al. 2007; G. G. Knyazev 2007). Therefore, the observed desynchronization likely reflects an enhanced attentional gain directed toward the movie's auditory channel, such as dialogue or emotional scores, during emotionally salient moments.

In the delta band, both subjective arousal and pupil diameter showed significant negative correlations with STG activity (Figure 7). This shared correlation suggests that delta suppression partly reflects basic sensory processing common to both measures, such as tracking the acoustic envelope (e.g., loudness or speech rhythm; Baltzell et al. 2016; Boucher et al. 2019; Bröhl and Kayser 2021; Horton et al. 2013). Furthermore, the pronounced relationship with arousal annotations aligns with top‐down attentional modulation: as the listener becomes more emotionally engaged with the narrative, they actively tune into behaviorally relevant auditory cues, such as the emotional prosody and affective tone of the dialogue (Hoekert et al. 2008; Koch et al. 2018). Thus, the delta desynchronization tied to arousal annotations indexes the active, attentive processing of the narrative's auditory emotional delivery, beyond just the raw acoustic input.

The cognitive demands of subjective reporting were further highlighted in the low IAF band, reinforcing the concept of ‘narrative immersion’. Arousal annotations exhibited a robust, source‐localized negative correlation with low IAF power that extended beyond the STG to include the middle temporal gyrus (MTG). This extended network involvement is highly consistent with the top‐down disinhibition required for higher‐order semantic integration and audiovisual processing of the narrative (Bonilha et al. 2017; Davey et al. 2016). While pupil diameter captures global autonomic activation and raw acoustic intensity (Gingras et al. 2015; Paulus et al. 2020), the pronounced source‐localized IAF tracking observed for subjective annotations specifically reflects the cognitive operations of ‘listening’ and comprehending the story.

Together, these findings support a cortical activation model where increased attention to the narrative leads to a broad desynchronization of low‐frequency rhythms (Hasson et al. 2004). This activation is not a uniform reflex to sound intensity but is specifically prioritized by the emotional relevance of the scene, recruiting the auditory cortex to process the semantic content of the unfolding narrative.

### 4.4 Precuneus Activation During Narrative Processing

In the precuneus we observed a significant arousal‐mediated desynchronization (cortical activation) in the low‐frequency bands (see Figure 7). To interpret this finding, it is necessary to situate the precuneus within its functionally heterogeneous architecture. Anatomically, the precuneus spans regions involved in externally goal‐directed attention (e.g., the superior parietal lobule, BA7) as well as internally directed cognition. For the latter, it serves as a central hub of the Default Mode Network (DMN), specifically, its Posterior Medial Network (PMN) subsystem, alongside the medial prefrontal cortex and hippocampus (Cooper et al. 2021; Cooper and Ritchey 2019).

Recent work deconstructing the PMN during naturalistic viewing has established a critical functional distinction: while the Ventral PMN is specialized for processing discrete event boundaries, the Dorsal PMN is responsible for the continuous tracking of movie content throughout an event (Cooper et al. 2021). The primary computational task of this network is the construction and maintenance of ‘situational models’ (Ranganath and Ritchey 2012; Reagh and Ranganath 2018). A situational model is a multidimensional mental representation that binds the visuospatial elements of a scene with its abstract conceptual significance (e.g., plot, history and emotional weight). Acting as a ‘narrative buffer’, the precuneus must constantly relate current sensory inputs to prior knowledge. Indeed, given its core role within the broader DMN, the precuneus is highly specialized for episodic memory retrieval, exhibiting rapid peak responses to access internal representations (Andrews‐Hanna et al. 2010; Sestieri et al. 2011).

High‐arousal moments (such as plot twists, sudden threats or emotional climaxes) represent periods of high ‘informational surprise’. These moments demand a dual cognitive response: they trigger a surge in externally goal‐directed attention to track rapid sensory changes (recruiting dorsal parietal regions) alongside a rapid updating of the internal situational model to maintain narrative coherence (recruiting PMN nodes). Therefore, the broad low‐frequency desynchronization we observed during high arousal likely reflects the simultaneous neural effort of these two processes. As narrative intensity peaks, the broad precuneus region shifts from a synchronized ‘idling’ state into a highly active mode to both capture salient external details and bind them into the evolving internal story.

### 4.5 Attentional Gating

Beyond the precuneus (source space, Figure 7), the robust negative correlation between arousal measures and low IAF power extended broadly across posterior sensor regions (Figure 6). This widespread parietal desynchronization indicates a heightened state of cortical activation (disinhibition) during emotionally salient moments, a pattern that aligns with the critical role of large‐scale attentional networks in naturalistic viewing.

We propose that this widespread parietal activation reflects a dynamic interplay between attentional subsystems required for narrative comprehension. In established models of attention and memory (Cabeza et al. 2011), ventral parietal regions (such as the Temporoparietal Junction) anchor the Ventral Attention Network (VAN), which facilitates bottom‐up attention. During high‐arousal epochs, the VAN detects salient external features, such as emotional scenes or sudden scene changes, which serve as cues to interrupt ongoing processing and trigger memory retrieval (Jääskeläinen et al. 2021). Simultaneously, the recruitment of the Dorsal Attention Network (DAN) within dorsal parietal cortices supports top‐down, goal‐directed recall to make sense of those cues.

Viewed alongside our source‐level precuneus findings, a cohesive mechanism emerges: arousal acts as a global control signal that desynchronizes posterior alpha rhythms, thereby ‘opening the gate’ for both the bottom‐up capture of salient narrative events (via the VAN) and the top‐down reconstruction of the narrative history (via the DAN and precuneus) required to seamlessly integrate them.

### 4.6 Limitations

Several limitations should be considered when interpreting these findings. First, while our source‐space analyses yielded theoretically consistent localizations (e.g., precuneus, superior temporal gyrus), EEG source estimation is inherently limited by spatial blurring and the ill‐posed inverse problem. Furthermore, because individual structural MRIs were not available for our cohort, source localization was performed using a standard template brain rather than subject‐specific anatomical models. Therefore, the precise anatomical boundaries of the active networks (particularly regarding adjacent temporal or parietal sub‐regions) should be interpreted as functional approximations rather than exact neuroanatomical mappings. Future studies integrating concurrent EEG‐fMRI or utilizing individual structural neuroanatomy, could definitively bridge this high‐temporal, high‐spatial divide. Second, our reliance on continuous subjective annotations inherently introduces a slight temporal lag. Unlike the instantaneous neural and autonomic reflexes captured by EEG and pupil diameter, continuously reporting arousal requires cognitive evaluation and motor execution. Third, while our results highlight distinct cortical networks prominently associated with subjective versus pupillary arousal, we did not formally test for a statistical interaction between these two continuous measures. Therefore, these network differences should be viewed as characteristic profiles rather than strict statistical dissociations. Finally, our final sample size (*N* = 25 for EEG) and our reliance on a single cinematic stimulus (Butterfly Circus) constrain the generalizability of our conclusions. While naturalistic viewing maximizes ecological validity, commercial films inherently conflate low‐level sensory transients (e.g., sudden loud noises or visual cuts) with high‐level narrative climaxes. Although our parallel tracking of subjective and pupillary responses helps disentangle this, further research utilizing diverse, independent cinematic samples is required to confirm whether these oscillatory arousal dynamics generalize across different emotional valences and cinematic genres.

## 5 Conclusion

This study demonstrates that emotional arousal during naturalistic viewing is a dynamic, brain‐wide phenomenon. By leveraging the highly synchronized behavioural and physiological responses of the audience to decode individual cortical oscillations, our multi‐level approach successfully mapped the neural architecture of narrative immersion. Our findings reveal that rather than a uniform reflex, narrative arousal relies on the orchestrated interplay of bottom‐up sensory gating and top‐down cognitive integration operating in tandem.

Specifically, posterior low‐frequency desynchronization demonstrated that pupillary dynamics closely track the bottom‐up enhancement of the sensory signal‐to‐noise ratio. Concurrently, continuous subjective reporting highlighted the top‐down cognitive demands of narrative evaluation. This active integration was reflected by fronto‐central theta activity, alongside broad low‐frequency desynchronization in the precuneus, a signature likely indexing the rapid retrieval of episodic memories required to update the situational model during plot shifts. Furthermore, low‐frequency suppression in the temporal cortices revealed that subjective arousal captures top‐down attentional modulation, reflecting an active tuning to semantic and emotional acoustic cues. Ultimately, by capturing these continuous neural dynamics, this work provides a time‐resolved, ecologically valid understanding of how the human brain processes complex emotional narratives in the real world.

## Author Contributions

**Magdalena Camenzind:** conceptualization, data curation, formal analysis, methodology, supervision, validation, visualization, writing – original draft, writing – review and editing. **Melanni Nanni‐Zepeda:** conceptualization, data curation, formal analysis, investigation, methodology, project administration, software, supervision, validation, visualization, writing – original draft, writing – review and editing. **Anna P. Giron:** data curation, investigation, project administration, software, supervision, writing – review and editing. **Konrad Dapper:** resources, writing – review and editing. **Michael Esterman:** writing – original draft, writing – review and editing. **Flavio Frohlich:** funding acquisition, resources, validation, visualization, writing – original draft, writing – review and editing. **Agnieszka Zuberer:** conceptualization, funding acquisition, methodology, project administration, resources, software, supervision, validation, visualization, writing – original draft, writing – review and editing.

## Funding

This work was supported by the Interdisciplinary Center for Clinical Research IZKF Tübingen.

## Conflicts of Interest

The authors declare no conflicts of interest.

## Supporting information


**Figure S1:** Objective data quality assessment and participant exclusion based on spatial rank. The bar plot illustrates the number of retained spatial data rank for each of the initial 32 participants following electrode interpolation and ICA‐based artefact rejection. To ensure sufficient spatial degrees of freedom for accurate and stable cortical source localization, an a priori quality threshold was established to retain at least 75% of the original spatial variance (derived from the 64‐channel setup). The red dashed line indicates this strict threshold at a rank of 48. Datasets requiring the rejection of more than 16 data ranks (typically reflecting continuous, uncorrectable muscle tension or movement artefacts throughout the movie) were deemed to have an insufficient signal‐to‐noise ratio for naturalistic EEG analysis. Based on this objective criterion, 25 participants (blue bars) were retained for the final analysis and 7 participants (red bars) were excluded.

## Data Availability

The datasets analysed during the current study are available from the corresponding author on reasonable request. Aggregated data and code are available on OSF: https://osf.io/75mzh/overview.
