# Supplementary figures and images for: Neural Oscillations Track Subjective and Pupillary Arousal During Naturalistic Movie Viewing
Source: Eur J Neurosci. 2026 May 12;63:e70543. doi: 10.1111/ejn.70543 (PMC13162759; doi:10.1111/ejn.70543)

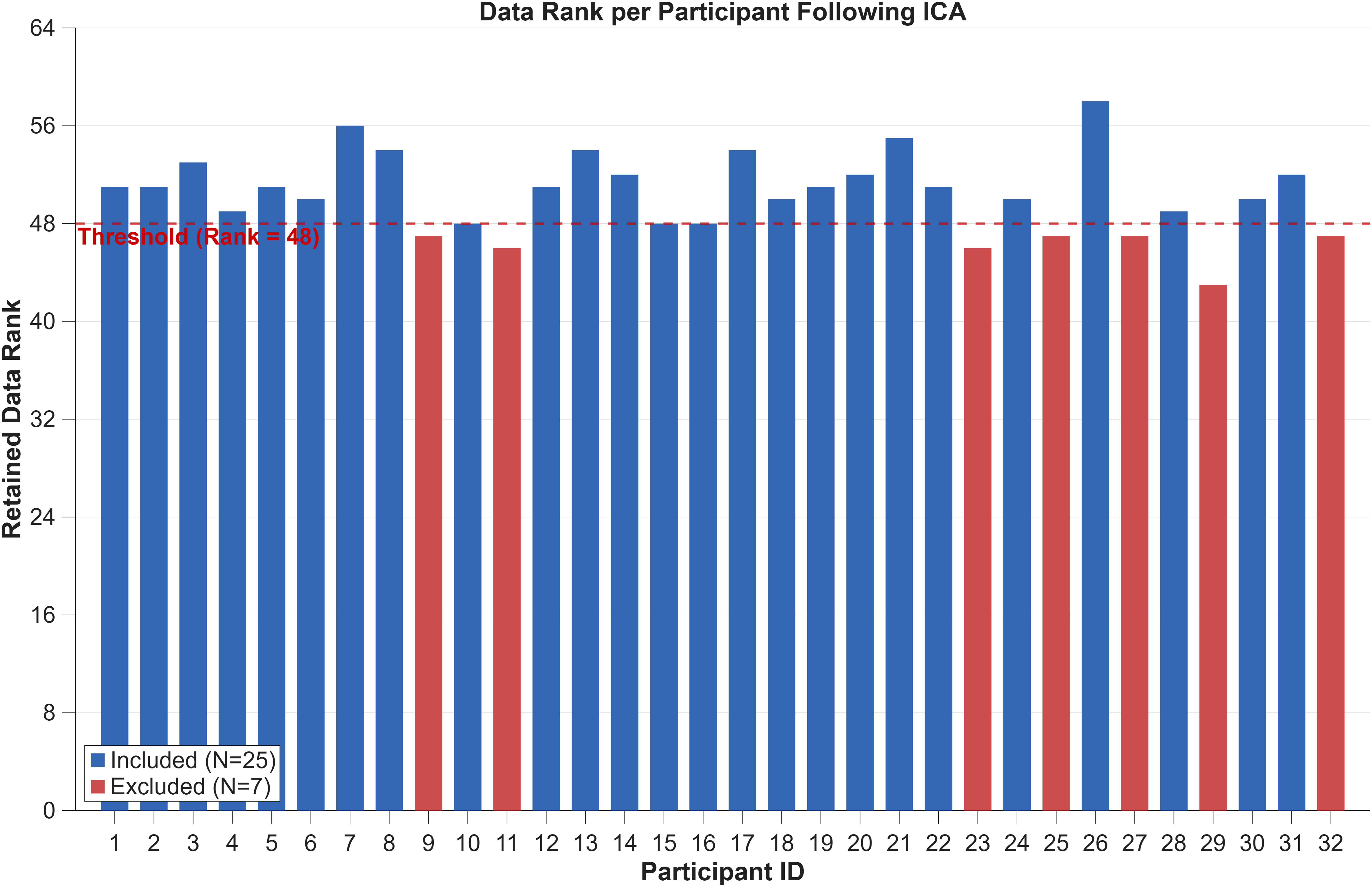

Supplement: Supplementary file 1 — Figure S1: Objective data quality assessment and participant exclusion based on spatial rank. The bar plot illustrates the number of retained spatial data rank for each of the initial 32 participants following electrode interpolation and ICA‐based artefact rejection. To ensure sufficient spatial degrees of freedom for accurate and stable cortical source localization, an a priori quality threshold was established to retain at least 75% of the original spatial variance (derived from the 64‐channel setup). The red dashed line indicates this strict threshold at a rank of 48. Datasets requiring the rejection of more than 16 data ranks (typically reflecting continuous, uncorrectable muscle tension or movement artefacts throughout the movie) were deemed to have an insufficient signal‐to‐noise ratio for naturalistic EEG analysis. Based on this objective criterion, 25 participants (blue bars) were retained for the final analysis and 7 participants (red bars) were excluded. [file EJN-63-0-s001.tiff]
